# Ethical Considerations in Human-Centered AI: Advancing Oncology Chatbots Through Large Language Models

**DOI:** 10.2196/64406

**Published:** 2024-11-06

**Authors:** James C L Chow, Kay Li

**Affiliations:** 1 Department of Radiation Oncology University of Toronto Toronto, ON Canada; 2 Princess Margaret Cancer Centre University Health Network Toronto, ON Canada; 3 Department of English University of Toronto Toronto, ON Canada

**Keywords:** artificial intelligence, humanistic AI, ethical AI, human-centered AI, machine learning, large language models, natural language processing, oncology chatbot, transformer-based model, ChatGPT, health care

## Abstract

The integration of chatbots in oncology underscores the pressing need for human-centered artificial intelligence (AI) that addresses patient and family concerns with empathy and precision. Human-centered AI emphasizes ethical principles, empathy, and user-centric approaches, ensuring technology aligns with human values and needs. This review critically examines the ethical implications of using large language models (LLMs) like GPT-3 and GPT-4 (OpenAI) in oncology chatbots. It examines how these models replicate human-like language patterns, impacting the design of ethical AI systems. The paper identifies key strategies for ethically developing oncology chatbots, focusing on potential biases arising from extensive datasets and neural networks. Specific datasets, such as those sourced from predominantly Western medical literature and patient interactions, may introduce biases by overrepresenting certain demographic groups. Moreover, the training methodologies of LLMs, including fine-tuning processes, can exacerbate these biases, leading to outputs that may disproportionately favor affluent or Western populations while neglecting marginalized communities. By providing examples of biased outputs in oncology chatbots, the review highlights the ethical challenges LLMs present and the need for mitigation strategies. The study emphasizes integrating human-centric values into AI to mitigate these biases, ultimately advocating for the development of oncology chatbots that are aligned with ethical principles and capable of serving diverse patient populations equitably.

## Introduction

### Overview

The development of oncology chatbots underscores the critical need for systems grounded in human-centered artificial intelligence (AI) principles that prioritize empathy, accuracy, and personalized patient support. In the context of oncology, where patients and their families often face significant emotional and informational challenges, these chatbots are essential tools for addressing their unique concerns [1-6]. However, as the adoption of large language models (LLMs) such as GPT-3 and GPT-4 becomes increasingly common in health care, the ethical considerations surrounding their use have grown in importance. It is vital that oncology chatbots adhere to ethical standards that ensure fairness, transparency, accountability, and respect for user privacy and autonomy. These systems should be designed to serve diverse user groups, particularly those from underrepresented communities, by avoiding biases and ensuring equitable treatment [7,8]. Human-centered AI in oncology focuses on creating systems that prioritize the needs and experiences of patients and health care providers, thereby enhancing care, empathy, and support. Ethical AI extends beyond mere technical functionality; it involves embedding principles that safeguard the well-being, dignity, and rights of patients. This includes building trust through transparency, securing patient data, and delivering accurate and bias-free information [9-12].

This review explores the integration of generative AI and LLMs into oncology chatbots, aiming to create tools that embody these human-centered AI principles. The customization and personalization of chatbots are essential to meet the specific needs of each user, transforming traditional chatbots from basic information providers into empathetic, patient-focused tools that significantly enhance the care experience [1,13,14]. The primary goal of this review is to examine the challenges and ethical concerns associated with deploying AI in sensitive health care settings, particularly oncology. As these technologies become more widespread, it is crucial to ensure that they align with human-centered ethical principles. This study is motivated by the need to address potential biases in AI systems, which could inadvertently harm the very patients they are designed to support.

The paper contributes to the field by identifying and analyzing key ethical challenges associated with oncology chatbots, with a specific focus on biases in the datasets used to train these models. Unlike previous studies that provide broad discussions on AI ethics, this review specifically addresses the unique ethical dilemmas faced in oncology, where the stakes are exceptionally high. The study also offers practical strategies for developers and health care providers to enhance the ethical development of AI, proposing a framework for human-centered AI in oncology. The findings of this study reveal that oncology chatbots often endure biases rooted in their training data, leading to unfair or ineffective outcomes. To address these issues, the paper provides strategic recommendations, such as using more diverse and representative datasets, implementing continuous monitoring, and refining training methodologies. These measures aim to ensure that AI-driven tools in oncology are not only effective but also ethically sound. In comparison to existing literature, this study offers a focused analysis of the ethical implications specific to oncology chatbots, an area that has been relatively underexplored. By providing a detailed examination of the sources of bias and presenting practical solutions, this paper advances the conversation on ethical AI in health care, particularly within the critical field of oncology.

### Enhancing Oncology Chatbots With Ethical and Human-Centered AI

In oncology, ethical principles like beneficence, nonmaleficence, autonomy, and justice are crucial to ensure patient well-being. Oncology chatbots, designed to support patients and families, must adhere to these guidelines. For example, a chatbot for patients with breast cancer can provide personalized treatment information and emotional support, ensuring that the information is accurate, culturally sensitive, and delivered with empathy [13]. Such chatbots can significantly ease the burden on patients by offering timely and relevant information. However, these chatbots also face ethical challenges, particularly in maintaining privacy and data security. For instance, a pediatric oncology chatbot must securely handle sensitive data, requiring robust encryption and transparent data usage policies [14]. Additionally, regular updates and monitoring are essential to prevent biases or inaccuracies that could harm patients. Transparency is another critical concern. Oncology chatbots must clearly disclose their AI nature to users. For instance, in end-of-life care, failing to inform users that they are interacting with an AI could lead to mistrust and harm the health care organization’s reputation. A proactive approach with clear self-disclosure at the start of interactions is essential to maintain trust [15]. In health care domains like nephrology, similar ethical considerations apply, with a focus on patient consent, privacy, and bias mitigation. In educational settings, oncology chatbots can also be valuable, but they must follow ethical frameworks to ensure accurate information delivery and fair AI operation. By adhering to these principles, oncology chatbots can effectively bridge learning gaps while maintaining trust and integrity [6,16-18].

### Designing Ethical and Trustworthy Oncology Chatbots With Human-Centered AI

Ethical chatbots, therefore, need to adhere to certain principles. They should prioritize transparency, providing users with clear indications when they are interacting with AI rather than a human. Respecting user privacy, obtaining informed consent, mitigating biases, ensuring data security, and promoting responsible AI use in education are central to developing ethical chatbots. By integrating ethical frameworks and considering societal impact, chatbots can contribute positively while upholding ethical standards in their interactions with users.

There are 2 concerns to build an ethical oncology chatbot in human-centered AI—first, it has to build trust in the users. Second, how can it build trust? Building a human-centered approach to AI-driven chatbots involves a strategic integration of several key elements. First, the design should prioritize the user’s needs and expectations. Rather than merely dispensing information mechanically, the chatbot should discern and address human needs relevantly. This personalized approach fosters a more trustworthy relationship between the user and the AI. Trust emerges as a pivotal concern in the development of AI chatbots.

Second, essential strategies can be used to cultivate trust in the oncology chatbots. The first involves personalization tailored to each user, enhancing the sense of individual relevance and reliability [19]. The second entails infusing the oncology chatbot with a human-like persona, creating a relatable and approachable interaction for users [20]. Implementing these qualities in the design of AI chatbots requires a thoughtful technical strategy. While the focus here is less on technical aspects and more on user experience and interaction, achieving personalization involves machine learning algorithms capable of understanding and adapting to individual user preferences [21]. Meanwhile, instilling a human-like persona necessitates sophisticated natural language processing (NLP) techniques and dialogue design that emulate human conversational patterns [22]. In essence, the development of human-centered AI chatbots revolves around creating an experience that seamlessly integrates technical prowess with an empathetic understanding of human needs through web-based inputs with the users. By bridging the gap between technological sophistication and human-like interaction, these chatbots can truly serve as effective companions in addressing users’ queries and needs.

### Ethical Challenges in Implementing Transformer-Based AI Models for Health Care Enhancement

One way is in the design of transformers. In 2019, transformers were used to create LLMs such as Bidirectional Encoder Representations from Transformers (BERT) and GPT-2 [23]. The integration of AI technologies, specifically LLMs, holds immense potential for improving efficiency and decision support in health care settings. However, ethical considerations become paramount when deploying such models, especially in critical domains like health care.

GPT-4, the underlying model of ChatGPT, has demonstrated significant potential in conversational AI applications [24]. This advancement has sparked discussions about the ethical implications of deploying such powerful models in health care. One primary concern is the potential inaccuracies in generated content. LLMs can produce convincing yet incorrect information, posing a risk of errors in medical records. Compounding this issue is the opacity of training data, making it challenging to assess accuracy effectively [25]. To address this concern, it is crucial for LLMs like GPT-4 to train on precise and validated medical datasets [26].

The growing integration of AI chatbots, exemplified by tools such as ChatGPT and Google Bard, in health care, introduces critical security implications [27,28]. While these AI-driven systems hold significant promise for improving patient care and public health, their reliance on massive datasets, including sensitive patient information, raises concerns about data security. During the pandemic, health care chatbots have become extensively used, addressing tasks like appointment scheduling and providing health information [29]. However, this increased usage magnifies security risks and privacy challenges that remain understudied. AI chatbots, like ChatGPT, also pose unique challenges in ensuring patient privacy and compliance with regulations such as the Health Insurance Portability and Accountability Act (HIPAA) [30]. Recent viewpoints in medical journals highlight the need for providers to navigate HIPAA compliance while safeguarding patient data [31]. Additionally, the safety of medical AI chatbots in patient interactions becomes a paramount consideration, necessitating measures to protect patient data, maintain information accuracy, and ensure user understanding [32]. Ethical considerations, including privacy and data security concerns, further complicate the widespread adoption of conversational AI in health care, emphasizing the need for comprehensive guidelines and robust encryption methods to build trust and safeguard sensitive health information in this era of AI-driven health care communication. Another critical ethical consideration is model bias [33]. LLMs may inadvertently perpetuate biases present in their training data, leading to medically inaccurate and discriminatory responses. Biases can stem from various sources such as sampling, programming, and compliance, necessitating careful consideration to avoid perpetuating harmful stereotypes. Striking a balance between model accuracy and unbiased responses is essential for responsible deployment in health care settings.

Privacy, a fundamental principle in health care, adds another layer of ethical complexity when using public LLMs. The potential risks associated with data sharing must be mitigated through strict agreements and HIPAA-compliant training protocols. Ensuring patient privacy is paramount in the implementation of AI technologies in health care [34].

Despite the potential benefits of using AI technologies, particularly transformer-based models, in health care, careful consideration of ethical principles is crucial. Addressing concerns related to accuracy, bias, and privacy will facilitate responsible and patient-centered implementation, benefiting both health care professionals and patients.

The insights from the Megatron transformer underscore the ethical considerations in deploying transformer models like ChatGPT [35]. Trained on vast datasets, Megatron suggests AI’s incapacity to independently ensure ethical behavior, emphasizing its tool-like nature dependent on human usage. Addressing the potential biases in transformer models, especially in health care, demands a focus on fairness metrics, proactive bias detection, and diverse training data. Continuous user feedback becomes crucial for iterative refinement, and bias-awareness training for stakeholders fosters a culture of ethical responsibility. Integrating these strategies into the deployment of transformer models is imperative, ensuring more equitable and inclusive AI-generated content across diverse applications.

### Ethical Considerations in Deploying LLMs in Health Care and Education

Using LLMs raises ethical considerations, including the potential for biased outputs, breaches of privacy, and the risk of misuse. These may have serious implications in medical settings. Addressing these concerns requires the adoption of transparent development practices, the responsible handling of data, and the integration of fairness mechanisms.

The integration of LLMs, such as ChatGPT, in medical practice and research raises crucial ethical issues concerning bias, trust, authorship, equitability, and privacy [32]. Although this technology has the potential to revolutionize medicine and medical research, being mindful of its potential consequences is essential. An outright ban on the use of this technology would be shortsighted. Instead, establishing guidelines that aim to responsibly and effectively use LLMs is crucial.

LLMs, like BioGPT [36] and LaMDA (Google Brain) [37], are currently under exploration for various applications in the medical field, showcasing versatility in tasks such as text generation, summarization, and aiding in clinical documentation and academic writing. The integration of LLMs, including oncology chatbots powered by ChatGPT, holds promise for streamlining essential health care tasks, including template creation, summarizing academic content, and enhancing the clarity of clinical notes. This potential introduces significant time-saving and efficiency gains in medical settings.

However, the incorporation of LLMs, particularly oncology chatbots, into health care applications also presents ethical challenges that demand careful consideration to ensure responsible use. Recent research underscores concerns related to the attribution of credit and rights for content generated by LLMs. Users may encounter difficulties in fully claiming credit for positive outcomes while potentially facing responsibility for unintended consequences, such as the generation of misinformation. This highlights the pressing need for updated perspectives on responsibility and the establishment of clear guidelines addressing issues like authorship, disclosure, educational applications, and intellectual property in the context of oncology chatbots and LLMs in general. Navigating the ethical implications of integrating oncology chatbots and LLMs into the medical field requires a comprehensive approach to foster responsible and transparent use of these powerful language models in health care settings.

In the field of education, LLMs show potential in automating tasks such as question generation, feedback provision, and essay grading. However, concerns about practicality and ethics, including technological readiness, transparency, and privacy considerations, must be addressed. A systematic scoping review identifies these challenges and recommends updating innovations with state-of-the-art models, open-sourcing models or systems, and adopting a human-centered approach in development. Therefore, the ethical considerations surrounding the use of LLMs in various fields in medicine and education, necessitate a careful and responsible approach. Establishing clear guidelines such as ensuring transparency and incorporating human oversight are essential steps in harnessing the benefits of LLMs while mitigating potential risks.

As a research group focused on human-centered AI and the ethical integration of AI principles into medical and oncology chatbots [1-6], particularly leveraging LLMs [32], our analysis delves into the historical evolution and the transformative potential of LLMs. We aim to spotlight the continuum of advancements in computational theory that has shaped our technological landscape, emphasizing the pivotal role of integrating humanistic and ethical considerations into AI for health care.

## LLMs and NLP Unveil New Potential for Human-Centered AI in Oncology Chatbots

### Neural Networks and Machine Learning

Neural networks, fundamental to modern AI, emulate the structure and functioning of the human brain, forming the basis for various applications [38]. In the medical context, the integration of LLMs like ChatGPT brings forth unprecedented possibilities. LLMs are part of the NLP domain and are built on architectures such as GPT and BERT [23,39]. Unlike rule-based models, LLMs learn unsupervised from extensive text data during pretraining, gaining a profound understanding of syntax, grammar, and context. Fine-tuning follows, adapting their knowledge for tasks like text generation and sentiment analysis.

Within the broader landscape of human-centered AI, the principles of neural networks and machine learning persist. The capacity of neural networks to capture complex patterns in data, combined with machine learning algorithms, remains instrumental. In the realm of human-centered AI, LLMs and NLP play a crucial role. NLP focuses on enabling machines to comprehend and generate human language, aligning with the principles of human-centered AI [40]. LLMs, as a significant advancement in NLP, excel in understanding and generating human-like language, enhancing natural interactions between AI systems and users. In the context of oncology chatbots, the integration of LLMs is pivotal. These advanced models empower chatbots to comprehend medical queries, respond empathetically, and adapt to diverse communication styles, ultimately improving the user experience in health care interactions. The use of LLMs in oncology chatbots not only fosters effective communication but also reinforces the human-centered aspect by creating more empathetic and context-aware interactions within the medical domain.

### LLMs in Oncology Chatbot

LLMs have achieved remarkable breakthroughs, innovating the field of NLP with their capacity to generate human-like text and excel in a multitude of NLP tasks. A compelling example is their application in the development of oncology chatbots [23,32,41]. These chatbots have the ability to communicate with users in a natural and coherent manner, offering invaluable assistance to both health care professionals and patients. LLMs have enabled oncology chatbots to generate human-like responses, providing users with a web-based and intuitive experience. These chatbots can understand complex medical queries, extract relevant information from patients’ descriptions of their symptoms, and generate responses that are not only accurate but also easily comprehensible to laypersons. This human-like text-generation capability significantly enhances the user experience, fostering trust and improving communication between patients and health care providers [42].

**Figure 1:**
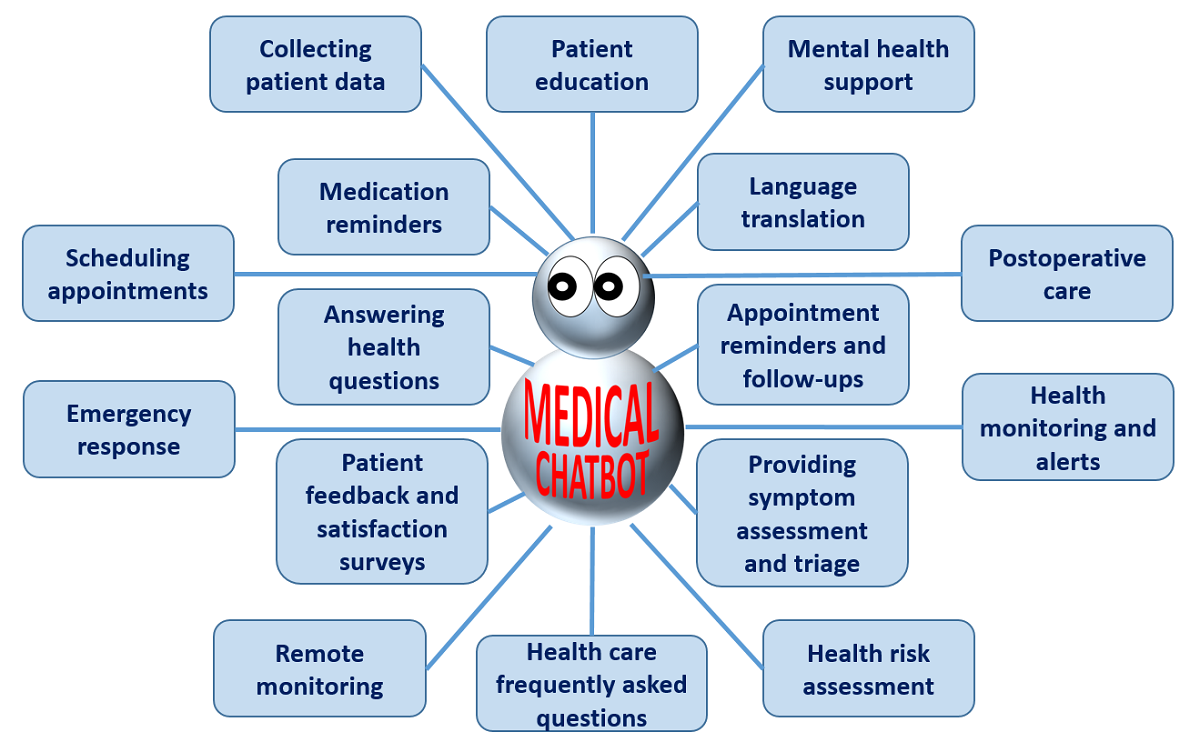
Various applications of an LLM-powered oncology chatbot. LLM: large language model.

Furthermore, LLMs empower oncology chatbots to perform diverse NLP tasks within the health care domain. They can extract critical information from medical records, assisting in patient diagnosis and treatment recommendations. These chatbots can also provide medication information, offer guidance on healthy lifestyles, and even support mental health through empathetic conversations [43,44]. Their versatility makes them invaluable tools in health care, augmenting the capabilities of medical professionals and providing accessible, round-the-clock health care information and support. Figure 1 shows the various applications of an oncology chatbot powered by LLMs.

## Applications and Implications of LLMs for Human-Centered AI in Oncology Chatbots

### Practical Applications of LLMs in Human-Centered AI

**Figure 2:**
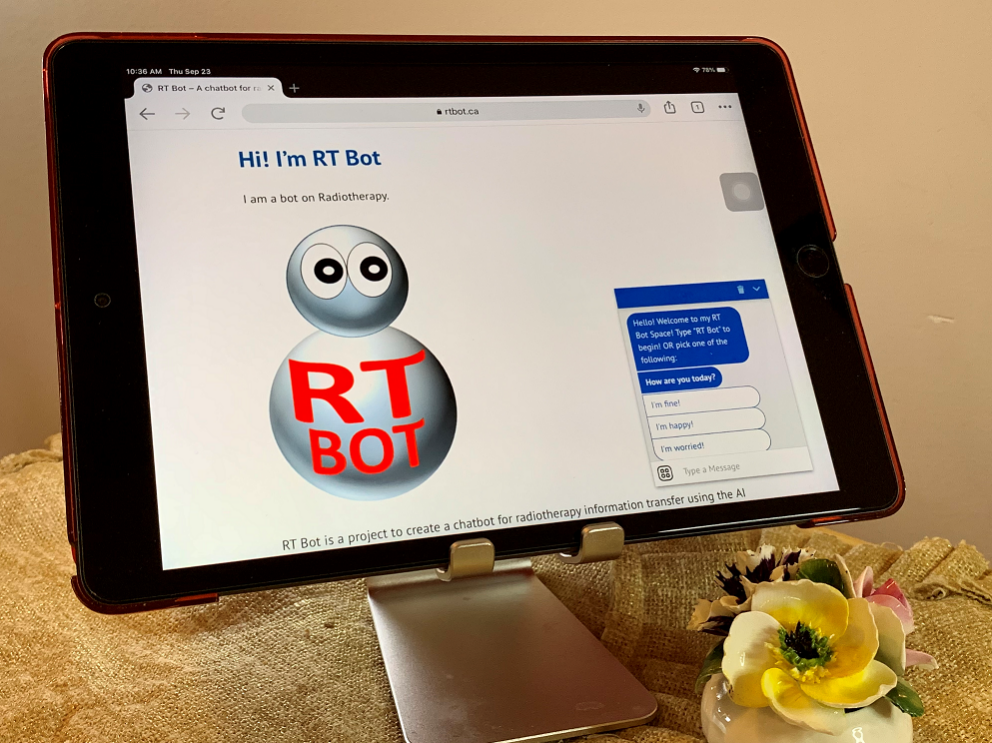
The RT Bot providing education in radiotherapy.

LLMs have demonstrated extensive practical applications across diverse domains, showcasing their versatility and transformative potential within the framework of human-centered AI. In the realm of language translation, these models have markedly enhanced the precision and fluency of machine translation systems [45]. They adeptly translate text among multiple languages, fostering seamless cross-cultural communication and bolstering global business operations. Within text generation, LLMs exhibit proficiency in crafting human-like text for multifarious purposes, aiding content creation by drafting papers, generating marketing copy, or assisting authors in producing creative content [46]. Moreover, LLMs find use in chatbots and web-based assistants, delivering natural and contextually sensitive responses in customer support, health care, and various other industries [47,48]. An illustration in Figure 2 depicts an example of the RT Bot used in radiotherapy education, epitomizing the integration of LLMs within the sphere of human-centered AI applications.

In software development, LLMs have demonstrated their prowess in code generation and code completion tasks. They can assist programmers by generating code snippets, fixing bugs, and enhancing productivity [49]. Moreover, in data analytics, LLMs are used for natural language querying of databases, simplifying data exploration and analysis for nontechnical users [50]. Moreover, LLMs are invaluable in the health care sector, where they aid in medical record analysis, diagnosis support, and drug discovery [51]. They can sift through vast amounts of medical literature to extract relevant information and assist health care professionals in making informed decisions. LLMs are also used in sentiment analysis and social media monitoring, helping businesses gauge public opinion, and adapt their strategies accordingly [52].

### Humanistic and Ethical AI

Humanistic AI refers to the approach in AI development that prioritizes human values, well-being, and understanding in the design and implementation of AI systems [53]. It emphasizes creating AI technologies that align with human principles, fostering empathy, compassion, and a deeper understanding of human needs and emotions. On the other hand, ethical AI involves adhering to moral principles and guidelines in the development and deployment of AI systems [54]. It encompasses considerations of fairness, transparency, accountability, privacy, and the societal impact of AI applications. Ethical AI aims to ensure that AI technologies benefit individuals and communities while minimizing potential harm or biases. Incorporating humanistic and ethical AI principles into oncology chatbots is crucial. Humanistic AI prioritizes empathy and understanding of human needs, while ethical AI ensures fairness, transparency, and accountability. This dual focus not only aligns with societal expectations but also safeguards against biases and harm, ensuring AI benefits individuals and communities in the medical domain [55-57].

The development of LLMs and oncology chatbots is deeply intertwined with the concepts of humanistic and ethical AI. LLMs, such as GPT-3 and GPT-4, are designed to generate human-like text and have been applied to various domains, including health care [58]. Oncology chatbots powered by LLMs aim to provide assistance, information, and even preliminary diagnosis to users [59]. Humanistic AI in oncology chatbots based on LLMs involves creating interfaces and interactions that are more empathetic, understandable, and accommodating to human emotions and concerns [60]. It seeks to imbue these AI systems with a human touch, making them more relatable and comforting for users seeking medical information or support. Ethical considerations in the development of LLM-based oncology chatbots are crucial. These AI systems must maintain patient privacy, ensure the accuracy and reliability of information provided, mitigate biases in data and responses, and offer transparent explanations for their suggestions or advice [61]. In addition, ethical AI in this context involves clearly delineating the capabilities and limitations of oncology chatbots to users, ensuring informed decision-making and responsible use of the technology.

Humanistic and ethical AI principles guide the responsible development and deployment of LLM-based oncology chatbots, promoting trust, reliability, and user satisfaction while addressing societal concerns and ethical implications associated with these AI-driven health care solutions [62].

### Societal and Ethical Implications of LLMs in Deploying Oncology Chatbots

The deployment of LLMs in the health care sector, particularly in the form of oncology chatbots, presents both significant benefits and ethical challenges. On one hand, oncology chatbots powered by LLMs can enhance access to health care information and provide quick assistance to users with medical queries. They offer a convenient means for individuals to seek information about symptoms, treatments, or health care recommendations. However, ethical concerns emerge when considering issues of privacy, security, and misinformation [63]. Oncology chatbots may inadvertently expose sensitive patient information if not properly secured, raising concerns about data breaches and privacy violations. Moreover, LLMs can potentially propagate medical misinformation, leading to incorrect self-diagnoses or treatment decisions [64]. The responsible development and deployment of oncology chatbots must involve robust data protection measures, continuous monitoring for accuracy, and adherence to medical ethics guidelines to ensure that these technological advancements contribute positively to health care while mitigating potential risks. Balancing the benefits of LLM-powered oncology chatbots with these ethical considerations is essential for their responsible and effective use in the health care domain [32]. Above all, the training datasets can be biased, and fall short of the underrepresented communities such as women, aboriginal people, persons with disabilities, and members of visible minorities [65,66]. The oncology chatbots still have to be trained to answer the needs of these communities.

## Challenges and Limitations

### Incorporating Humanistic and Ethical Principles Into LLM-Driven Oncology Chatbots

**Figure 3:**
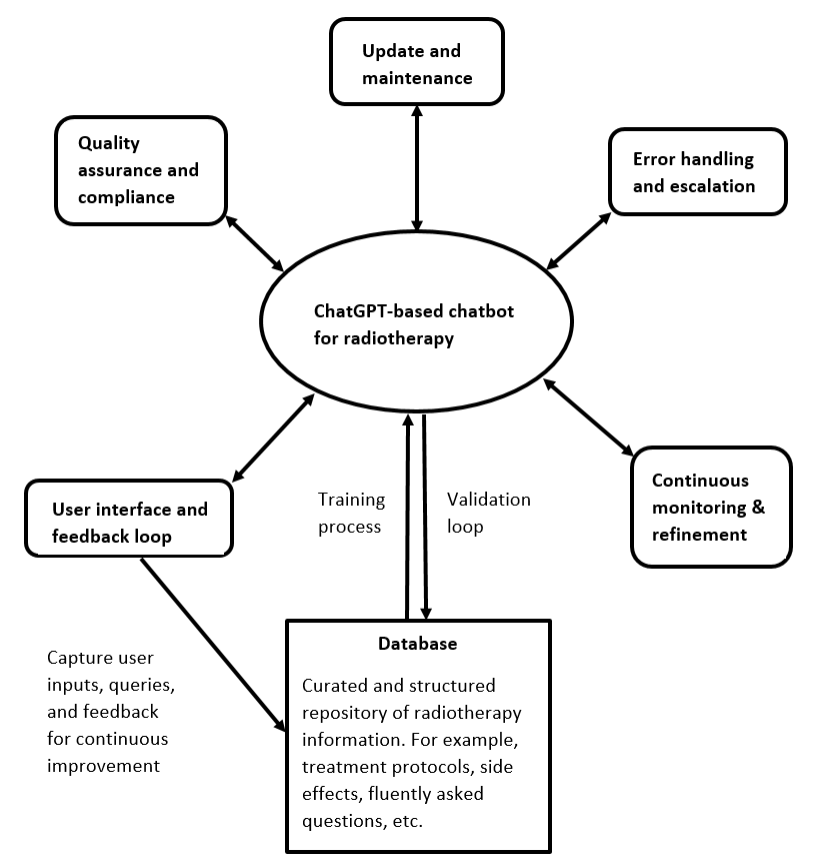
Schematic diagram showing the framework of medical chatbot based on large language model–based ChatGPT, focused on radiotherapy, ensuring accuracy, compliance, and continuous refinement.

The application of LLMs in oncology chatbots not only presents a promising avenue for enhancing health care accessibility and support but also introduces critical ethical considerations within the realm of human-centered AI [20]. Despite the potential benefits, the integration of these systems raises significant ethical concerns and safety considerations. One prevalent issue pertains to the potential perpetuation of biases and discrimination within these AI systems. LLMs, learning from extensive datasets that may inherently contain societal biases, risk generating skewed recommendations or responses that could adversely affect specific demographics, thus perpetuating health care disparities [67,68]. Moreover, the deployment of AI-driven chatbots might inadvertently impede individuals’ autonomy, recourse, and rights by overshadowing or dismissing their unique health care needs or preferences [69]. Transparency also remains a significant challenge, as these models often generate outcomes that are nontransparent, difficult to explain, or seemingly unjustifiable, making it challenging for users to comprehend or challenge the decisions made by the AI [32]. Furthermore, there are concerns regarding user privacy breaches, as personal health information shared with these chatbots may not always be adequately secured [70]. Additionally, the reliance on AI-driven interactions might risk isolation and the deterioration of the patient-doctor relationship, potentially undermining the crucial social connections essential for holistic health care [71]. Ensuring the reliability and safety of outcomes produced by these chatbots remains a concern, as inaccuracies or poor-quality responses could have detrimental consequences on patient health and well-being [72,73]. Mitigating these ethical challenges and ensuring the safety of LLM-based oncology chatbots necessitate robust frameworks, stringent regulations, and ongoing scrutiny to address potential harms and uphold ethical standards within the domain of human-centered AI in health care. Figure 3 shows a proposed framework of a radiotherapy chatbot based on ChatGPT. The chatbot is anchored by a robust core powered by ChatGPT, interfacing seamlessly with a meticulously curated database of verified medical information [74]. The model undergoes domain-specific training to enhance its comprehension of radiotherapy intricacies, while a continuous feedback loop ensures that validated data inform its responses and are cross-verified for accuracy. To enhance ethical AI practices, the framework should incorporate bias mitigation strategies by diversifying data sources, ensuring transparency about the chatbot’s capabilities and limitations, implementing robust user privacy measures, establishing continuous ethical reviews, providing user education on verifying information, and creating accessible feedback mechanisms for reporting inaccuracies. This iterative approach fosters a dynamic, reliable, and ethically responsible ecosystem for delivering accurate and up-to-date information within the scope of radiotherapy [75].

Therefore, integrating humanistic and ethical principles into LLM-based oncology chatbots stands as a significant challenge in contemporary AI development [76]. Achieving this integration requires a comprehensive approach. First, prioritizing patient confidentiality and data security remains pivotal. Implementing robust encryption measures and stringent access controls can effectively mitigate risks associated with sensitive medical information [77]. Second, infusing empathy and sensitivity into the chatbot’s responses poses a significant hurdle. It necessitates the development of algorithms capable of understanding and empathetically responding to patients’ emotional states, demanding extensive research into sentiment analysis and contextually appropriate language generation [78]. Moreover, carefully considering the ethical implications of decision**-**making in medical scenarios is crucial. Collaborative efforts among AI developers, ethicists, and medical professionals are vital to embed ethical guidelines into the chatbot’s algorithms, ensuring alignment with medical ethics and patient welfare [14,79,80]. Striking a balance between technical functionality and ethical considerations is key to fostering trust and acceptance of LLM-based oncology chatbots in the health care ecosystem. Continuous vigilance, ongoing refinement, and transparent communication about the chatbot’s capabilities and limitations are essential steps in responsibly integrating humanistic and ethical principles into this advancing technology [63,81].

### Approaches to Mitigate Bias in LLM-Driven Oncology Chatbots

To avoid potential bias in LLM-based oncology chatbots, it is crucial to adopt a comprehensive approach. First, ensure that the training data are diverse and representative of the entire population the chatbot aims to assist. This involves incorporating information from various demographic groups, ethnicities, genders, and socioeconomic backgrounds to prevent the model from learning and perpetuating biases present in specific subsets of data [82,83]. Moreover, ethical data collection practices should be a priority, with developers implementing strict guidelines to eliminate unintentional biases. Transparently communicate ethical standards to users and stakeholders to foster trust and accountability in the development process [84]. Incorporating bias detection and correction algorithms during both the training and deployment phases is essential [85,86]. These mechanisms should be designed to identify and rectify biased outputs in real time, with regular updates to adapt to evolving data and user interactions. In addition, transparency is key in addressing bias; therefore, the chatbot should be designed to provide clear explanations for its decisions. This not only enhances user trust but also enables health care professionals to understand the reasoning behind the chatbot’s recommendations. Continuous monitoring and evaluation are also important to the chatbot’s success [87,88]. Regularly assess its performance over time, ensuring that potential biases are identified and corrected promptly. User feedback integration further enhances the system, allowing diverse user groups to report biases and contribute to ongoing improvements [1,2]. Furthermore, collaboration with health care professionals is paramount. Involving experts in the development and validation processes helps refine the chatbot’s responses, ensuring accuracy and minimizing biases that may arise from a lack of medical context [32]. Finally, regulatory compliance with health care and data protection standards is vital. Adhering to established regulations ensures that the chatbot operates within ethical and legal boundaries, building trust among users and health care providers alike [89]. Table 1 summarizes the strategies for mitigating bias in LLM-based oncology chatbots.

**Table 1:** Strategies for mitigating bias in large language model–based oncology chatbots.

Strategies	Description
Diverse and representative training data	Use data that reflect the diversity of the target population
Ethical data collection practices	Implement strict ethical guidelines for data collection
Bias detection and correction algorithms	Integrate algorithms to identify and correct biased outputs
Explainability and transparency	Design the chatbot to provide clear explanations for decisions
User feedback integration	Continuously monitor and evaluate the chatbot’s performance
Collaboration with health care professionals	Encourage user feedback to identify and address biases
Privacy-preserving models	Involve health care experts in development and validation
Regulatory compliance	Adhere to health care and data protection regulations

### Navigating the AI Frontier: Challenges and Ethical Considerations

The rise of LLMs, exemplified by GPT-4, has sparked both excitement and apprehension. Geoffrey Hinton, a prominent figure in deep learning, acknowledges their potential to surpass human intelligence [90]. However, this rapid progress raises ethical and safety concerns. Despite having significantly fewer connections than the human brain, LLMs exhibit remarkable learning capabilities. Their ability to generalize from limited examples challenges conventional wisdom. Hinton argues that their occasional errors and hallucinations are features, akin to human imperfections. His fears extend beyond mere intelligence; he emphasizes the risk of AI misuse by malicious actors. Whether in elections or warfare, AI’s capacity to create subgoals and manipulate environments demands urgent attention. Responsible development and regulation are imperative. Hinton envisions a hybrid intelligence—a fusion of learning and communication—where machines outperform humans in both domains. This transformative era requires collective action and societal discussions akin to historical agreements on chemical weapons. As AI development outpaces regulation, Hinton questions whether our existing social structures can handle the implications. Responsible AI deployment necessitates interdisciplinary collaboration and thoughtful governance. While some may dismiss Hinton’s concerns, the stakes are high. As we navigate the path toward AI advancement, we must grapple with the potential consequences and strive for ethical, human-centered progress.

### Concerns of Datasets in LLMs or NLP for Ethical and Human-Centered AI

The ethical considerations surrounding the datasets used in training LLMs and NLP systems are critical for advancing human-centered AI, particularly in the context of oncology chatbots. The datasets used for training these models often reflect societal biases, which can lead to ethical dilemmas when the outputs of these chatbots are applied in real-world health care settings [57]. For instance, commonly used datasets like the Common Crawl, Wikipedia, and clinical databases may overrepresent affluent, Western demographics while underrepresenting minority groups, non-Western cultures, and marginalized communities. This bias can result in oncology chatbots that are less effective in serving diverse patient populations, potentially exacerbating health disparities. Moreover, the ethical implications of dataset bias become evident when examining specific LLMs like GPT-3 and GPT-4. These models are often fine-tuned on domain-specific datasets, which can inadvertently amplify existing biases. For example, if an oncology chatbot is trained predominantly on datasets from high-income health care systems, it may lack the cultural competency required to address the needs of patients from low-income or diverse backgrounds [91]. Such a scenario not only limits the chatbot’s effectiveness but also raises concerns regarding equity in health care delivery. Table 2 outlines how to address the concerns related to datasets in LLM or NLP for ethical and human-centered AI, specifically in the context of oncology chatbots. This table provides examples of datasets, identifies potential biases, and suggests strategies for mitigating these biases. By systematically addressing these issues, we seek to illuminate the vital importance of ethical dataset selection and its influence on developing effective, human-centered oncology chatbots. Our findings underscore the need for continuous evaluation and modification of datasets to reduce bias, ensuring that LLMs accurately represent and serve the diverse populations they aim to support in the field of oncology.

**Table 2:** Overview of dataset, potential biases, and strategies for mitigation in large language model or natural language processing medical chatbot.

Dataset	Description	Potential biases	Strategies for mitigation
Common Crawl	A large dataset collected from web pages across the internet	Overrepresentation of Western cultures, socioeconomic status	Ensure diverse sourcing and include localized health care data from various regions
Wikipedia	Open-source encyclopedia with content generated by volunteers	Gender and racial biases due to contributor demographics	Use guidelines for inclusive contributions and diversify contributor base
MIMIC-III	Critical care database with deidentified health data	Predominantly includes data from urban hospitals; underrepresents rural populations	Incorporate data from a variety of health care settings, including rural and underserved areas
Health-related Twitter data	Tweets related to health topics used for sentiment analysis	Possible bias in language and topics relevant to affluent groups	Filter and include tweets from diverse socioeconomic backgrounds and global populations
Clinical trials data	Data from clinical trials used to evaluate treatments	Limited representation of minority groups in trial participants	Prioritize inclusion of diverse populations in future trials and datasets
PubMed studies	Biomedical literature and research papers	Predominantly Western-centric studies may neglect non-Western medical practices	Integrate literature from diverse geographical regions and cultural contexts
Patient health records	Deidentified patient data for training models	Disparities in data collection practices may overlook marginalized groups	Standardize data collection practices to ensure comprehensive representation

## Future Directions

While LLMs have undoubtedly showcased remarkable capabilities, it is crucial to recognize their inherent limitations, especially when applied in the context of oncology chatbots. One of the most pronounced constraints is the absence of genuine understanding [92]. LLMs excel at producing coherent and contextually relevant text, yet they lack true comprehension or reasoning abilities. In the realm of oncology chatbots, this limitation can manifest in responses based solely on patterns from their training data, without a deep grasp of medical principles [93]. Furthermore, there is the risk of unintentionally generating misleading or inaccurate content, a particularly critical concern in health care, where erroneous information can carry significant consequences [94]. Therefore, the deployment of oncology chatbots should be approached as a supplementary aid alongside human medical professionals [95]. It is imperative to navigate their limitations thoughtfully while maintaining vigilant oversight to ensure the precision and reliability of the information they furnish.

Ongoing research in the realm of LLMs is dedicated to confronting their limitations and enhancing their reliability, interpretability, and ethical standing. One particularly promising avenue focuses on the development of more resilient training datasets that seek to mitigate bias and encompass a broader spectrum of perspectives and languages [96]. Researchers are actively exploring methods to render LLMs more interpretable, facilitating users in comprehending and trusting their decision-making processes. Additionally, there is a mounting emphasis on ethical considerations, including the establishment of guidelines and regulations governing LLM deployment, content generation, and the protection of data privacy [97]. Upholding transparency, accountability, and fairness in LLMs is fundamental to their responsible use. Future directions may encompass the creation of hybrid models that combine the strengths of LLMs with other AI techniques, ultimately enhancing their reliability while diminishing the likelihood of generating misleading information [98]. As LLMs assume an increasingly central role across diverse domains, ongoing research and ethical considerations are pivotal forces shaping their development and deployment for the betterment of society.

Future directions for LLMs focus on several key areas of advancement. These include enhancing the models’ ability to understand context, nuances, and user intent in natural language, which will lead to more effective human-computer interactions. There is also a growing emphasis on integrating text-based models with vision and audio capabilities, enabling richer and more comprehensive communication. Addressing and reducing biases in LLMs is critical to ensuring fairness and inclusivity in generated content, while customization and fine-tuning of models are becoming increasingly important for specific applications. Efforts are also being made to develop more energy-efficient LLM architectures and training methods, which would reduce their environmental impact and make them accessible on low-power devices. Real-time conversational AI is another area of focus, with the goal of enabling more seamless, natural, and context-aware interactions. Privacy-preserving models are being researched to protect user data, and human-AI collaboration is being advanced to enhance productivity and creativity. Ethical guidelines and regulations are being established to ensure the responsible and safe use of LLMs [99]. In education, LLMs are being used to create personalized and adaptive learning experiences. In the medical field, these models are expanding their role in research, diagnostics, and patient care, with a strong emphasis on adhering to medical ethics and ensuring compliance with standards such as patient confidentiality and informed consent. Finally, the creative capabilities of LLMs are being explored, pushing the boundaries in generating content across various artistic domains.

The integration of LLMs into the domain of oncology chatbots raises intriguing opportunities and concerns, underscoring the significance of human-centered AI within health care applications. While LLMs offer a powerful tool for enhancing human-computer interactions, particularly in health care settings, their application necessitates careful consideration and balance [100,101]. Historically, expert systems have played a pivotal role in decision support and knowledge representation within these applications. The incorporation of LLMs introduces a novel dimension to this landscape by capitalizing on their remarkable capacity to comprehend and generate human language. However, it is crucial to recognize that akin to expert systems, LLMs possess inherent limitations. While excelling at processing extensive data and generating coherent responses, their actual grasp of intricate medical principles may be constrained. Therefore, the primary challenge lies in harnessing the capabilities of LLMs while ensuring that their responses align with medical accuracy, ethical considerations, and the ethos of human-centered AI in health care.

## Conclusions

The emergence of LLMs signifies a transformative leap in computational paradigms, highlighting the central role of human-centered AI in this progression. Models such as GPT-3 and GPT-4 have not only revolutionized machine learning but have also profoundly influenced oncology chatbots through their advanced language processing capabilities. However, as technological advancements persist, the ethical dimensions—particularly concerning biases and misinformation—require meticulous attention. Integrating humanistic and ethical principles into the development of LLMs, especially within oncology chatbots, is crucial for responsible AI integration. Envisioning a future where machines possess unparalleled language abilities alongside adept management of ethical complexities demands a proactive ethical framework.

This comprehensive review explores the evolution, applications, and future trajectories of LLMs in health care and beyond. It is essential to acknowledge the inherent limitations and dynamic nature of technology, suggesting that the landscape of LLMs is rapidly evolving. Future directions outlined herein may witness significant changes or novel developments shortly. Therefore, ongoing research efforts should continuously update and expand this review, encompassing newer LLM iterations, exploring specific health care applications, and conducting empirical studies to validate practical implications and real-world efficacy.

Furthermore, deeper exploration into the ethical implications and societal impacts of widespread LLM implementation remains a critical avenue for future inquiry. Continued research endeavors in these areas will not only enhance our comprehension and use of LLMs but also address emerging challenges and opportunities, aligning with the foundational principles of human-centered AI.

## Abbreviations

AI: artificial intelligence
BERT: Bidirectional Encoder Representations from Transformers
HIPAA: Health Insurance Portability and Accountability Act
LLM: large language model
NLP: natural language processing
